# Elucidation of blink reflex characteristics in Parkinson's disease subtypes through prepulse inhibition

**DOI:** 10.3389/fneur.2025.1505598

**Published:** 2025-05-07

**Authors:** Zhen Zhang, Ling Zhang, Xiaofeng Huang, Xinqing Hao, Tao Li, Yayin Luo, Xiaoxue Yin, Chunli Song, Zhanhua Liang

**Affiliations:** Department of Neurology, The First Affiliated Hospital of Dalian Medical University, Dalian, China

**Keywords:** Parkinson's disease, prepulse inhibition, blink reflex, postural abnormal gait disorder, tremor-dominant, lateralization

## Abstract

This study investigated prepulse inhibition (PPI), a brainstem reflex, in Parkinson's disease (PD) patients. We compared PPI impairment between patients with postural instability and gait difficulty (PD-PIGD) and tremor-dominant (PD-TD) subtypes and explored potential lateralization effects. Fifty PD patients and 35 healthy controls underwent pre-pulse stimulation of the finger followed by stimulation of the supraorbital nerve. Compared to controls, PD patients exhibited impaired PPI across various stimulation intervals, with a more pronounced effect in the PD-PIGD subgroup. Interestingly, no significant differences in PPI were observed between the left and right sides, suggesting a bilateral effect. These findings suggest that abnormal brainstem circuits, potentially involving the pontine nucleus, contribute to PPI dysfunction in PD. Furthermore, the association between impaired PPI and the PD-PIGD subtype highlights a potential link with gait disturbances. Future research could explore the utility of PPI as a biomarker for gait dysfunction and treatment response in PD.

## 1 Introduction

Parkinson's disease (PD) is a progressive neurological disorder that causes movement problems and other issues. China faces a significant and growing burden due to PD, with an estimated 2 million patients currently diagnosed (1). This number is projected to rise dramatically as the population ages, potentially reaching 5 million within the next 25 years. This surge will impose a substantial strain on social resources. Therefore, a thorough understanding of the underlying neural mechanisms of PD is crucial. This knowledge will pave the way for improved clinical diagnosis, more effective treatment options, and ultimately, a better life quality for PD patients.

PD presents with a distinct set of motor symptoms commonly encountered in clinical practice, such as resting tremor, rigidity, bradykinesia, and postural and gait abnormalities (2). Additionally, patients may experience a range of non-motor symptoms, including attention difficulties, cognitive decline, and autonomic dysfunction (3). Notably, clinical PD classification often relies on the type of movement disorders observed. One of the most widely adopted classification methods for PD is based on the work of Jankovic et al. (4). This system utilizes the ratio of average tremor score to average postural gait abnormality score derived from the Unified Parkinson's Disease Rating Scale (UPDRS). It categorizes patients into three groups: tremor-dominant (TD), postural instability and gait difficulty (PIGD), and the mixed type (5). Research suggests that PD primarily arises from the progressive loss of dopamine-producing neurons in a brain region called the substantia nigra. This loss disrupts the function of the basal ganglia motor circuit. Among PD subtypes, patients with PIGD symptoms is characterized by rapid disease progression, poor prognosis, and a reduced response to dopamine replacement therapy. Due to these characteristics, the PIGD subtype has sometimes been referred to as “malignant Parkinson's disease” (4, 6). In contrast to patients with PIGD symptoms, those with PD presenting primarily with resting tremors exhibit a relatively slower disease progression, a more favorable prognosis, a better response to conventional internal medicine treatment, and relatively fewer non-motor symptoms (4). Studies have shown that cognitive decline and attention deficits only manifest in tremor-dominant PD when accompanied by symptoms like postural instability and abnormal gait (4, 6).

Given the intractable nature of PIGD-PD patients, deep brain stimulation (DBS) of the globus pallidus or subthalamic nucleus has become a common clinical approach. However, this intervention only provides short-term symptom relief, and its long-term effectiveness, particularly for frozen gait, remains suboptimal. Promising new research suggests that deep stimulation of pedunculopontine tegmental nucleus (PPTN), also known as the dorsal tegmental nucleus (PPTg), may be a viable option for patients experiencing gait difficulties associated with PD. PPTN may affect movement, startle reflex, arousal state, and other functions. Notably, its role in gait suggests its potential as a key component of the midbrain motor area (7, 8).

The startle reflex is a well-known involuntary motor response to sudden, intense stimuli like loud noises, electric shocks, or unexpected touches (9). Humans typically experience this reflex as a blink, while animals exhibit a more generalized motor response (10). Prepulse inhibition (PPI) is a phenomenon where the weaker stimulus presented 30 to 500 ms before the main one significantly reduces the startle response (9). In the blink reflex, PPI specifically suppresses the amplitude of R_2_ wave, while shorter intervals between the prepulse and startle stimulus lead to increased R1 amplitude. PPI is considered a valuable model for studying cross-modal sensory gating, a process by which irrelevant sensory information is filtered out (9). Previous studies have established the impaired PPI as a characteristic feature of various mental illnesses, including schizophrenia and obsessive-compulsive disorder (10, 11). More recently, researchers have observed PPI deficits in motor disorders such as PD (12), cervical dystonia (13), and eyelid spasms (blepharospasm) (9). Previously, our team investigated the mechanism underlying impaired sensory motor gating in primary blepharospasm patients using PPI. We found that healthy individuals exhibited the most significant PPI at 200 ms, while patients with blepharospasm displayed impaired PPI. These findings suggest a potential dysfunction in brainstem regulation among blepharospasm patients.

To gain a more comprehensive understanding of PPI in the healthy Chinese population, we enlarged the control group sample size and extended the prepulse stimulation interval (ISIs) to 500 ms. This allowed us to perform a more detailed analysis of PPI behavior across various ISIs in PD patients in comparison to healthy controls. Furthermore, we investigated potential differences in PPI between two PD subtypes: tremor-dominant (TD) and postural instability and gait difficulty (PIGD) types. This study's findings contribute to understanding the neurophysiology of sensory motor gating deficits in PD. These insights may be applicable for guiding treatment strategies, developing new biological markers, and improving prognosis evaluation.

## 2 Materials and methods

### 2.1 Subjects

Our study involved participants from the Movement Disorders Clinic at the First Affiliated Hospital of Dalian Medical University. Fifty patients diagnosed with Parkinson's disease (PD) were enrolled, adhering to the Movement Disorder Society (MDS) criteria (14). Gender- and age-matched healthy controls (HC) included 35 participants. The ethical approval was provided by the Ethics Committee of our University [identification number: PJ-KS-2023-319(X)]. All subjects have written informed consent before participating.

We excluded potential participants with co-morbidities known to affect prepulse inhibition (PPI) to ensure the integrity of the data, such as schizophrenia spectrum disorders and temporal lobe epilepsy with psychosis (15). Comprehensive information of participants was gathered, including complete medical and family history, current medications, and for women, details about contraceptive use and menstrual cycle (16). Additionally, all participants were instructed to abstain from smoking and caffeinated beverages for at least 3 h before the experiment.

For the PD group, additional assessments were conducted. All participants completed the Unified Parkinson's Disease Rating Scale (UPDRS) to evaluate the motor symptoms. The testing in PD patients was conducted in the ON medication state. Levodopa equivalent daily dose (LEDD) was calculated using a standardized formula, taking into account their various anti-Parkinson's medications (17). Patients with PD were further grouped into postural instability and gait difficulty (PIGD), tremor-dominant (TD), and mixed subtypes based on the established UPDRS scoring system (5). The classification criteria involved comparing the mean tremor score to the mean postural instability and gait difficulty score. A ratio of 1.5 or greater for TD items to PIGD items indicated a TD classification. Conversely, a ratio of 1 or less was classified as PIGD. Patients with a ratio between 1 and 1.5 fell under the mixed subtype category.

### 2.2 Methods

Surface electromyography (EMG) was employed to capture the electrical activity of the muscles. We utilized a Synergy system (CareFusion, London, UK) for this purpose. The EMG recordings were filtered with a bandpass of 30–3,000 Hz to isolate the relevant signal components. Additionally, a high sampling rate of 2,000 Hz was used to ensure accurate capture of the rapid muscle responses. Prior to the experiment, participants received a detailed explanation of the various types of stimuli they would encounter. Importantly, the researcher and the equipment were positioned out of sight to eliminate the possibility of participants visually anticipating the stimulus type. This ensured unbiased responses during the experiment.

#### 2.2.1 Blink reflex (baseline trials)

**Figure 1:**
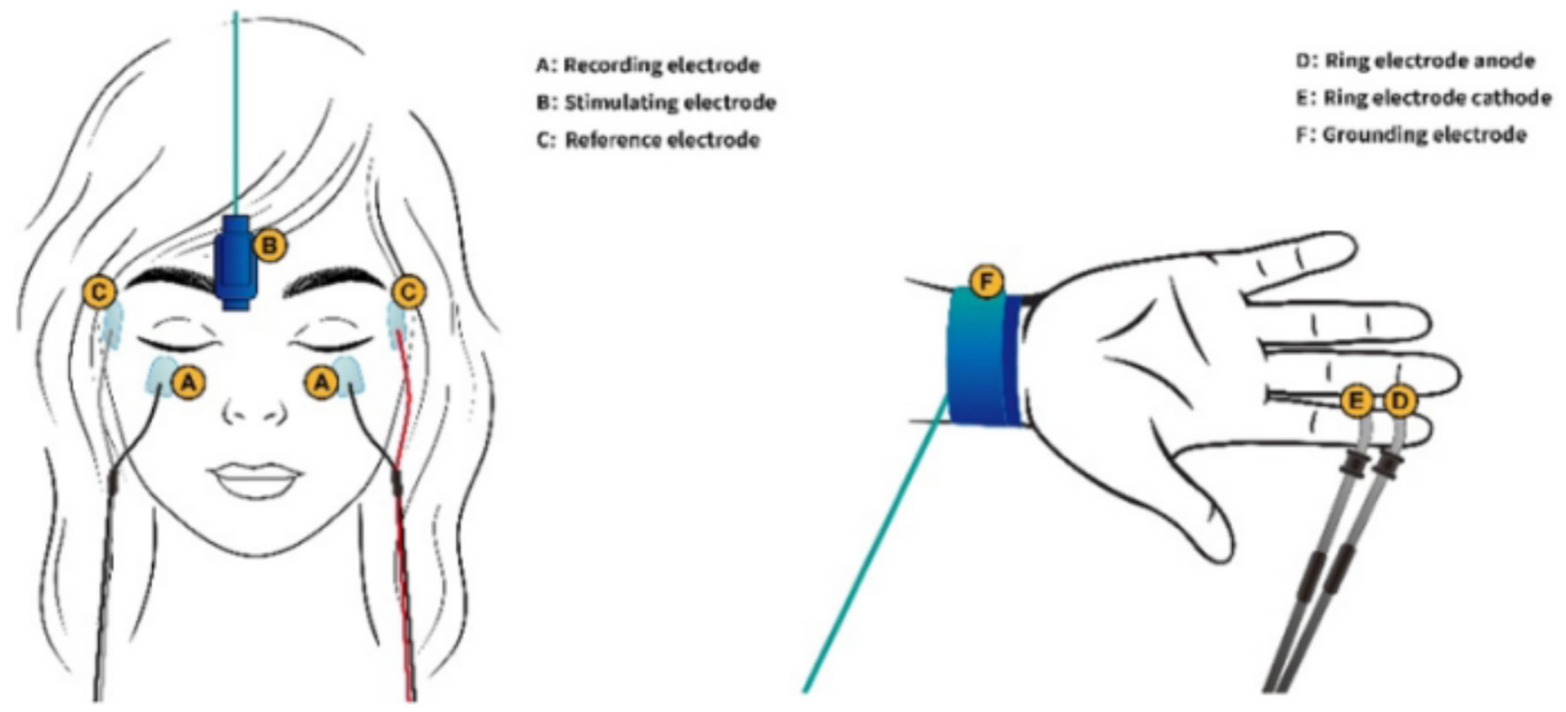
A, Recording electrode; B, Stimulating electrode; C, Reference electrode; D, Ring electrode anode; E, Ring electrode cathode; F, Grounding electrode.

For optimal comfort and data collection, participants were positioned in a supine (lying flat on their back) posture. They were instructed to gently close their eyes and relax. To record the orbicularis oculi muscle electrical activity (responsible for blinking), each blink reflex was elicited through surface electrodes, involving the electrodes on the lower eyelids, the reference ones 2 cm sideways from the outer corner of each eye, and the grounding one on the right wrist. We utilized constant current rectangular pulses delivered percutaneously above the right supraorbital notch (Figure 1). We set the pulse duration at a precise 0.2 milliseconds. To ensure a consistent and reliable response, the stimulus intensity was set at 10 times the sensory threshold. The threshold was determined as the lowest level of stimulus intensity detectable by the participant in at least 4 out of 8 trials. This calibration process ensured that all participants received a strong enough stimulus to elicit a blink reflex without causing discomfort.

#### 2.2.2 Prepulse inhibition

The prepulse stimuli were delivered at four different interstimulus intervals (ISIs)−120 ms (PPI_120_), 200 ms (PPI_200_), 300 ms (PPI_300_), and 500 ms (PPI_500_)—before the supraorbital nerve stimulation. These timings were chosen based on established research findings in this field. Similar to the main stimulus, the prepulse utilized a brief (0.2 milliseconds) electrical current delivered in a rectangular pattern. Ring electrodes attached to the middle and distal phalanges of the right index finger administered these prepulses (Figure 1). The prepulse intensity was carefully adjusted to be twice the sensory threshold, ensuring that participants could perceive the prepulse but that it was not uncomfortably strong. Four blink reflexes were elicited in each trial. The experiment incorporated a mix of baseline trials (without a prepulse) and prepulse trials, presented in a random order. To prevent anticipation, a randomized interval of 15–25 seconds was maintained between each trial.

## 3 Statistical analysis

Our analysis excluded trials containing artifacts. For each valid trial, we identified the key components of the blink reflex: the ipsilateral R_1_ wave (first response), the ipsilateral R_2_ wave (larger secondary response), and the contralateral R_2c_ wave (response in the opposite eye). The area under the curve (AUC) was used to quantify the R_2_ and R_2c_ component magnitude in each blink reflex. Subsequently, the following aspects of the blink reflex were determined: Ipsilateral R_1_ latency (time from stimulus to first response), Ipsilateral R_1_ peak-to-peak amplitude (maximum voltage difference), Bilateral R_2_ latencies (time from stimulus to secondary response in each eye), and AUC of bilateral R_2_ waves. Our primary outcome measure was the percentage change in R_2_ area, reflecting the magnitude of the PPI effect (hereafter, PPI size). We calculated this using the following formula: PPI size (in %) = [1 – R_2_ area at prepulse trials (120, 200, 300, or 500 ms)/R_2_ area at baseline trials] × 100%. Data was analyzed using IBM SPSS version 26.0. Prior to analysis, we assessed data normality using the Shapiro-Wilk test.

The following statistical tests were applied: Independent-sample or paired-sample *t*-test for normally distributed data, Chi-square test for categorical data like gender, and Wilcoxon rank-sum test or Mann-Whitney *U* test for non-normal data (e.g., some prepulse stimulus measures). H-Y staging scores, representing disease severity in PD patients [mean ± standard deviation (SD)]. Multi-group comparison was conducted using one-way ANOVA followed by *post hoc* test. All results are presented as mean ± SD. A *p*-value < 0.05 represents significant difference. Importantly, any significant findings were further corrected using the False Discovery Rate (FDR).

## 4 Results

### 4.1 Clinical data

To assess potential confounding factors, the demographic and clinical characteristics was first examined among the participants. Both age and gender distribution exhibited no significant differences between the Parkinson's disease (PD) and healthy control (HC) groups (Table 1). This ensures that any observed differences in the electrophysiological measures are likely due to the disease state rather than demographic factors. Following established criteria, PD patients were further categorized into two subgroup types: postural instability and gait difficulty (PD-PIGD) and tremor-dominant (PD-TD). The analysis showed no significant differences in both clinical characteristics and demographics between these subgroups (Table 2). This suggests that the subsequent electrophysiological assessments were conducted on well-matched PD subgroups.

**Table 1:** Demographic and clinical characteristics of the HC and PD groups.

	**HC (*n =* 35)**	**PD (*n =* 50)**	***p***
Age (years)	63.1 ± 12.0	65.6 ± 7.8	0.237
Gender M/F *n* (%)	15/20 (42.9%/57.1%)	27/23(54%/46%)	0.380
Hoehn-Yahr (H-Y) staging	-	2.2 ± 0.5	-
PD-TD *n* (%)	-	20(40%)	-
PD-PIGD *n* (%)	-	18(36%)	-

Statistical comparisons were conducted using the Mann-Whitney *U* test for non-normally distributed and qualitative data, and the independent-samples *t*-test for normally distributed data. Gender was compared using the Chi-square test (*p*-value reported).

**Table 2:** Demographic and clinical characteristics of the PD-PIGD and PD-TD groups.

	**PD-TD (*n =* 20)**	**PD-PIGD (*n =* 18)**	***p***
Age (years)	66.3 ± 8.9	63.1 ± 6.2	0.202
Gender M/F *n* (%)	9/11(45%/55%)	11/7(61%/39%)	0.352
Hoehn-Yahr (H-Y) staging	2.1 ± 0.5	2.3 ± 0.5	0.251
MDS-UPDRS III	26.2 ± 7.2	29.6 ± 5.5	0.133

Statistical comparisons were conducted using the Mann-Whitney *U* test for non-normally distributed and qualitative data, and the independent-samples *t*-test for normally distributed data. Gender was compared using the Chi-square test (*p*-value reported).

### 4.2 PPI in the PD and HC groups

**Figure 2:**
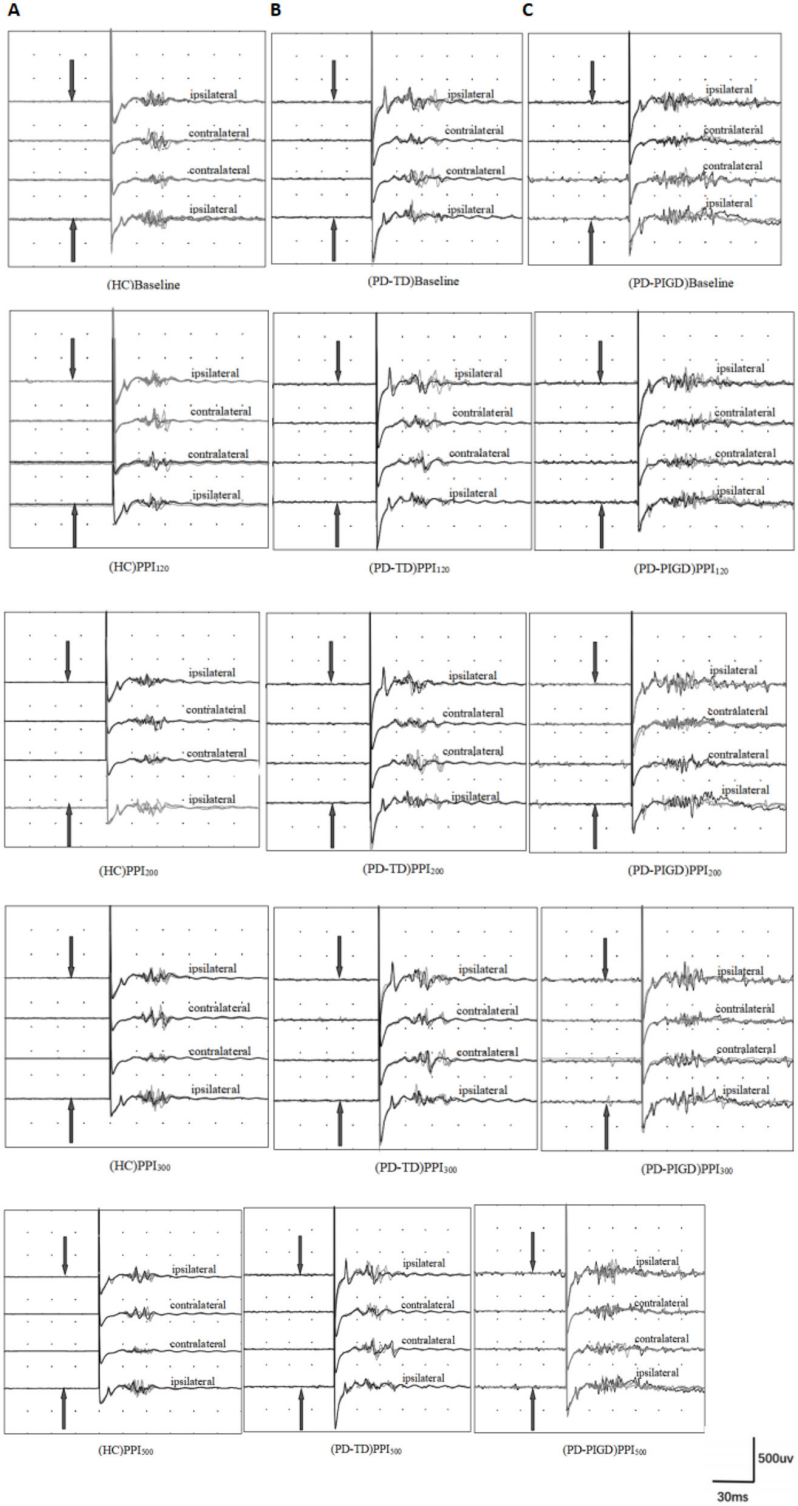
Prepulse inhibition of the blink reflex in healthy controls (HC) and Parkinson's disease (PD) groups, including postural instability gait disorder (PD-PIGD) and tremor-dominant (PD-TD) subtypes. Panels show results for: **(A)** HC group, **(B)** PD-TD group, and **(C)** PD-PIGD group. PPI_120_, PPI_200_, PPI_300_, and PPI_500_ represent prepulse inhibition at interstimulus intervals (ISIs) of 120 ms, 200 ms, 300 ms, and 500 ms, respectively. The top four traces depict the baseline blink reflex without a prepulse stimulus. The bottom 16 traces display the blink reflex following a prepulse stimulus (indicated by the arrow pointing to the index finger). Each trace is an average of four blink reflexes. PD patients demonstrated larger R_2_ and R_2c_ areas post-prepulse stimulation compared to HCs, with the PD-PIGD subgroup showing more significant R_2_ and R_2c_ areas than the PD-TD subgroup. This indicates that PD patients have impaired prepulse inhibition, with PD-TD patients having relatively better PPI than PD-PIGD patients.

**Figure 3:**
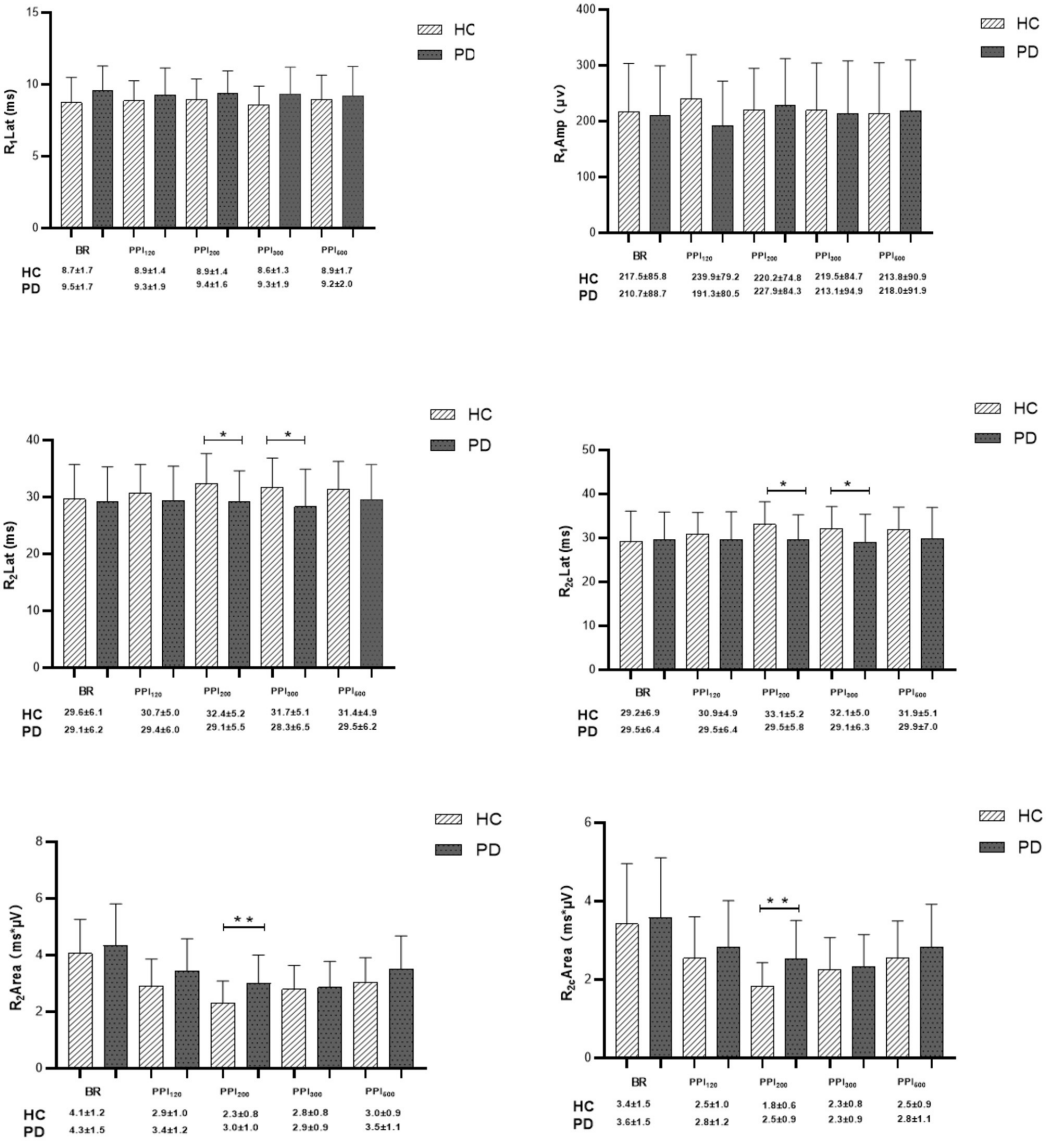
Comparison of blink-reflex neurophysiological data between healthy controls (HC) and Parkinson's disease (PD) groups. BR represents the baseline blink reflex without a prepulse stimulus. PPI_120_, PPI_200_, PPI_300_, and PPI_500_ denote prepulse inhibition at inter-stimulus intervals (ISIs) of 120 ms, 200 ms, 300 ms, and 500 ms, respectively. Differences in R_1_ latency, R_1_ amplitude, R_2_ latency, R_2c_ latency, R_2_ area, and R_2c_ area were determined between PD and HC groups at baseline and across various ISIs with prepulse trials. Qualitative data were analyzed using the Mann-Whitney *U*-test, and normally distributed data were analyzed using the independent-sample *t*-test. *p < 0.05, **p < 0.01 indicates significant changes after FDR correction.

Building upon the established demographic equivalences between groups, we explored the prepulse inhibition (PPI) response in the PD and HC groups. While bilateral supraorbital and prepulse stimulation was administered to all participants, blink reflex parameters at baseline and during prepulse stimulation with different interstimulus intervals (ISIs) were analyzed using data from the left eye in both the PD and HC groups (Figure 2). This approach simplifies data analysis without compromising the validity of the findings. At baseline (without prepulse stimulation), the PD group exhibited a longer R1 latency (time from stimulus to first response) compared to the HC group (Figures 2, 3). Additionally, we determined the area under the curve (AUC). The PD group displayed a slightly larger AUC for both the R_2_ and R_2c_ components of the blink reflex (Figures 2, 3).

**Figure 4:**
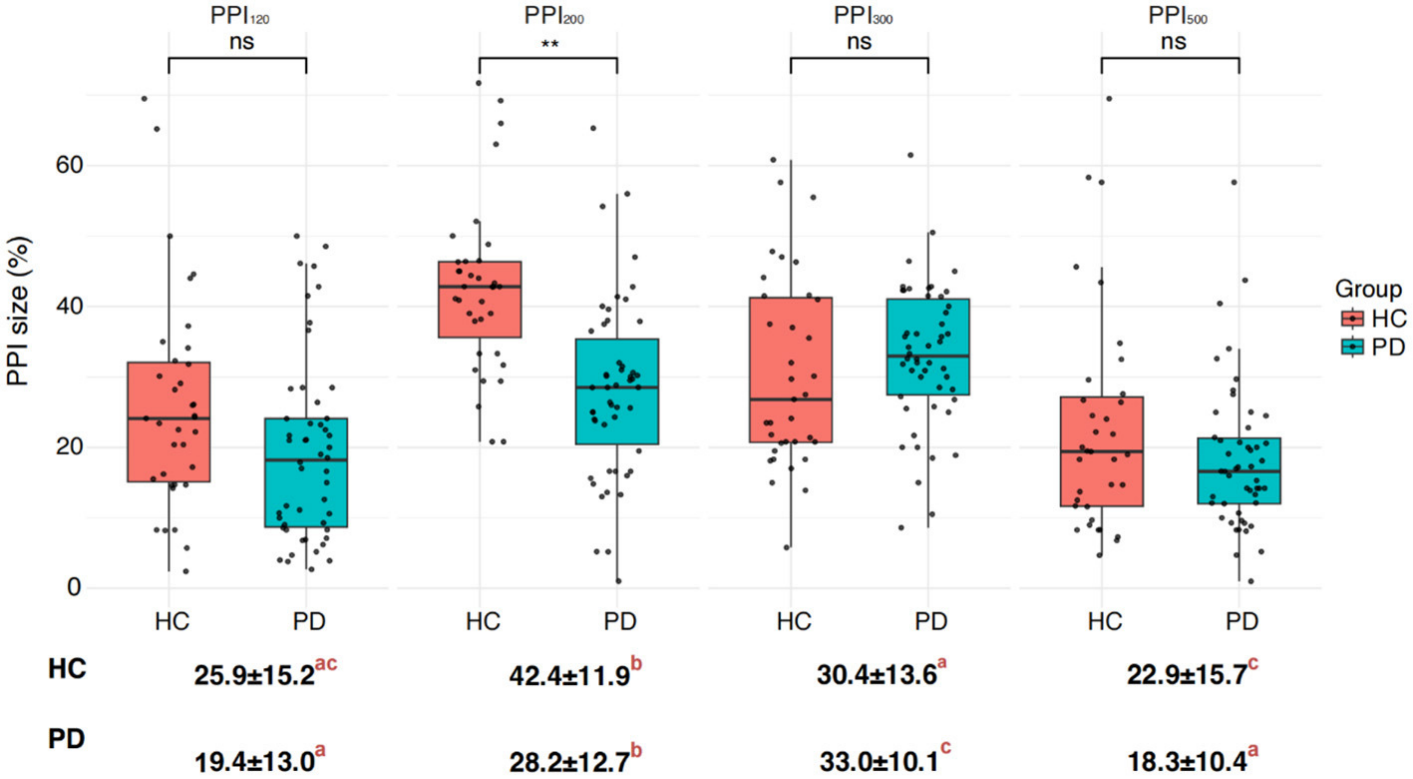
Differences in PPI size between the Parkinson's disease (PD) and healthy control (HC) groups. PPI_120_, PPI_200_, PPI_300_, and PPI_500_ represent prepulse inhibition at interstimulus intervals (ISIs) of 120 ms, 200 ms, 300 ms, and 500 ms, respectively. Comparisons between two groups at each ISI were performed using the independent-sample *t*-test. **p < 0.01 indicates significant changes after FDR correction. Letters denote significant differences between different ISIs within each HC or PD group, analyzed using one-way ANOVA followed by *post hoc* tests (*p* < 0.05).

In the HC group, prepulse stimulation resulted in a prolonged latency of the R_2_ and R_2c_ waves, reaching a maximum at 200 ms ISI (Figure 3). This effect indicates successful prepulse inhibition. Additionally, all four ISIs caused a decrease in the bilateral R_2_ area (Figures 3, 4), further supporting effective PPI. In contrast to HC, the PD group showed no significant increase in R_2_ and R_2c_ latency with prepulse stimulation. Moreover, the reduction in the bilateral R_2_ area (PPI) was less pronounced in the PD group (Figures 3, 4). These findings suggest a significantly decreased PPI in the PD patients compared to healthy controls.

Interestingly, prepulse stimulation at 120 ms ISI led to an increase in R_1_ amplitude in HC (Figure 3). The R_1_ amplitude peaked at 200 ms ISI in the PD group, but it did not reach the same level as observed in the HC. Overall, the PD patients displayed a consistently higher R_1_ latency in comparison to the healthy controls, with the most significant difference observed at baseline (Figure 3; Tables 3, 4). Furthermore, the difference in PPI size was statistically more pronounced at PPI_200_ compared to PPI_120_, PPI_300_, and PPI_500_ between the HC and PD groups (Figure 4).

**Table 3:** Neurophysiological differences in blink reflex at baseline and prepulse trials within the healthy control (HC) group.

**HC**	**Baseline**	**PPI_120_**	***p***	**FDR-q**	**PPI_200_**	***p***	**FDR-q**	**PPI_300_**	***p***	**FDR-q**	**PPI_500_**	***p***	**FDR-q**
R1 Lat	8.7 ± 1.7	8.8 ± 1.4	0.589	0.589	8.9 ± 1.4	0.514	0.589	8.6 ± 1.3	0.477	0.589	8.9 ± 1.7	0.321	0.589
R1Amp	217.5 ± 85.8	239.9± 79.2	0.053	0.212	220.2 ± 74.8	0.821	0.883	219.4 ± 84.7	0.878	0.883	213.8 ± 90.9	0.883	0.883
R2 Lat	29.6 ± 6.1	30.7 ± 5.0	0.006	**0.008** ^**^	32.4 ± 5.2	0.000	**0.000** ^**^	31.7 ± 5.1	0.000	**0.000** ^**^	31.4 ± 4.9	0.020	**0.020** ^*^
R2c Lat	29.2 ± 6.9	30.9 ± 4.9	0.031	**0.031** ^*^	33.1 ± 5.2	0.000	**0.000** ^**^	32.1 ± 5.0	0.002	**0.004** ^**^	31.9 ± 5.1	0.007	**0.009** ^**^
R2 Area	4.1 ± 1.2	2.9± 0.9	0.000	**0.000** ^**^	2.3 ± 0.8	0.000	**0.000** ^**^	2.8 ± 0.8	0.000	**0.000** ^**^	3.0 ± 0.9	0.000	**0.000** ^**^
R2c Area	3.4 ± 1.5	2.5 ± 1.1	0.000	**0.000** ^**^	1.8 ± 0.6	0.000	**0.000** ^**^	2.3 ± 0.8	0.000	**0.000** ^**^	2.5 ± 0.9	0.000	**0.000** ^**^

PPI_120_, PPI_200_, PPI_300_, and PPI_500_ represent prepulse inhibition at inter-stimulus intervals (ISIs) of 120 ms, 200 ms, 300 ms, and 500 ms, respectively. Non-normally distributed data were analyzed using the Wilcoxon rank-sum test, while normally distributed data were analyzed using the paired-sample *t*-test. ^*^p < 0.05 ^**^p < 0.01 indicate significant changes after FDR correction.

**Table 4:** Neurophysiological differences in blink reflex at baseline and prepulse trials within the Parkinson's disease (PD) group.

**PD**	**Baseline**	**PPI_120_**	***p***	**FDR-q**	**PPI_200_**	***p***	**FDR-q**	**PPI_300_**	***p***	**FDR-q**	**PPI_500_**	***p***	**FDR-q**
R1 Lat	9.5 ± 1.7	9.3 ± 1.9	0.741	0.741	9.4 ± 1.6	0.349	0.465	9.3 ± 1.8	0.247	0.465	9.3 ± 1.9	0.184	0.465
R1 Amp	216.7 ± 88.7	191.3 ± 80.5	0.332	0.664	227.9 ± 84.3	0.124	0.496	213.1 ± 94.9	0.783	0.783	218.0 ± 91.9	0.654	0.783
R2 Lat	29.1 ± 6.2	29.4 ± 6.0	0.637	0.849	29.1 ± 5.5	0.963	0.963	28.3 ± 6.6	0.299	0.849	29.5 ± 6.2	0.548	0.849
R2c Lat	29.5 ± 6.4	29.5 ± 6.4	0.893	0.992	29.5 ± 5.8	0.990	0.992	29.1 ± 6.3	0.992	0.992	29.9 ± 7.0	0.290	0.992
R2 Area	4.3 ± 1.5	3.4± 1.2	0.000	**0.000** ^**^	3.0 ± 1.0	0.000	**0.000** ^**^	2.9 ± 0.9	0.000	**0.000** ^**^	3.5 ± 1.2	0.000	**0.000** ^**^
R2c Area	3.6 ± 1.5	2.8 ± 1.2	0.000	**0.000** ^**^	2.5 ± 1.0	0.000	**0.000** ^**^	2.3 ± 0.8	0.000	**0.000** ^**^	2.8 ± 1.1	0.000	**0.000** ^**^

PPI_120_, PPI_200_, PPI_300_, and PPI_500_ represent prepulse inhibition at inter-stimulus intervals (ISIs) of 120 ms, 200 ms, 300 ms, and 500 ms, respectively. Non-normally distributed data were analyzed using the Wilcoxon rank-sum test, while normally distributed data were analyzed using the paired-sample *t*-test. ^**^p < 0.01 indicate significant changes after FDR correction.

### 4.3 PPI in the PD-TD and PD-PIGD groups

**Figure 5:**
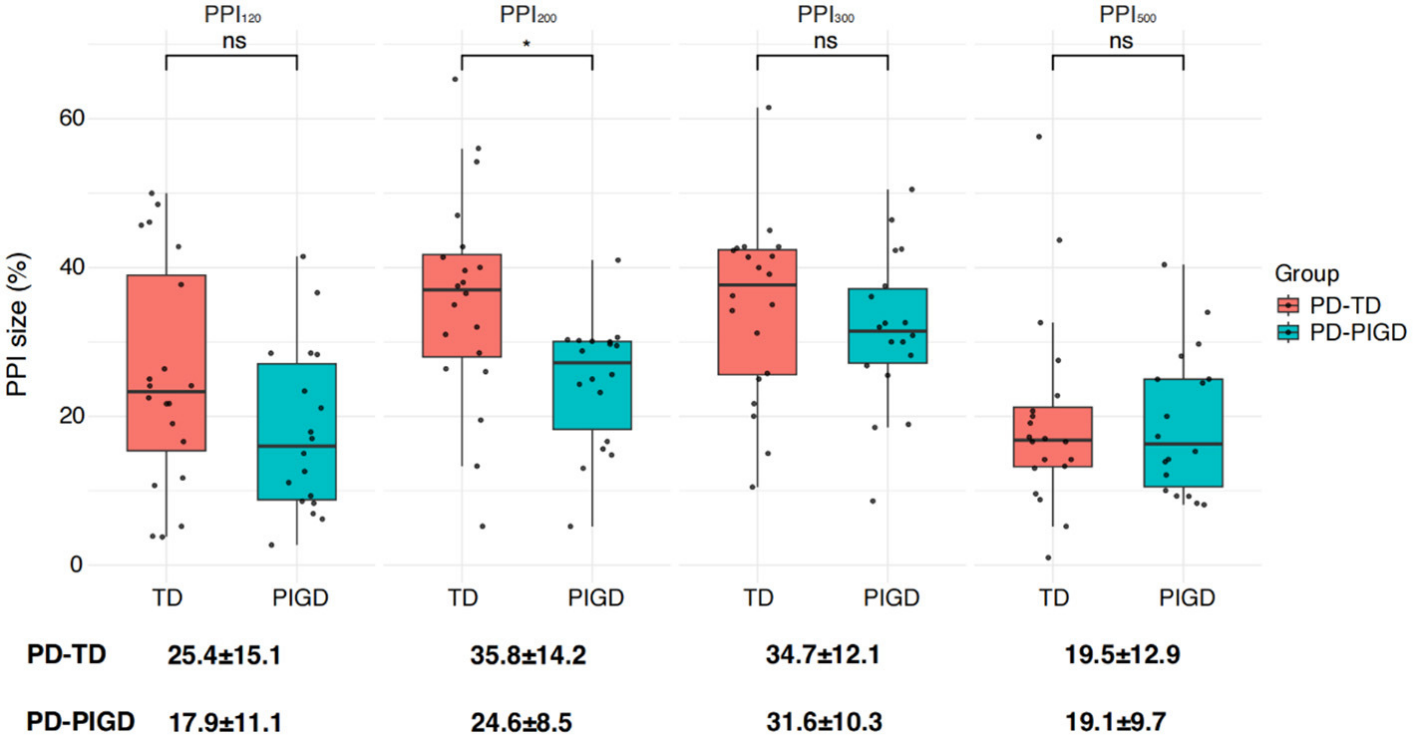
Differences in PPI size between the two Parkinson's disease subtypes: postural instability gait disorder (PD-PIGD) and tremor-dominant (PD-TD). PPI_120_, PPI_200_, PPI_300_, and PPI_500_ represent prepulse inhibition at interstimulus intervals (ISIs) of 120 ms, 200 ms, 300 ms, and 500 ms, respectively. Comparisons between two groups at each ISI were performed using the independent-sample *t*-test. *p < 0.01 indicates significant changes after FDR correction.

Following the comparison of overall group differences, we further examined PPI within the PD subgroups: tremor-dominant (PD-TD) and postural instability and gait difficulty (PD-PIGD) (Figure 5). Our findings revealed a higher level of PPI in the PD-TD subgroup in comparison to the PD-PIGD subgroup (Figure 5). This suggests that the PD-TD group displayed a stronger inhibitory response to the prepulse stimulation.

### 4.4 No lateralization of PPI effects

**Figure 6:**
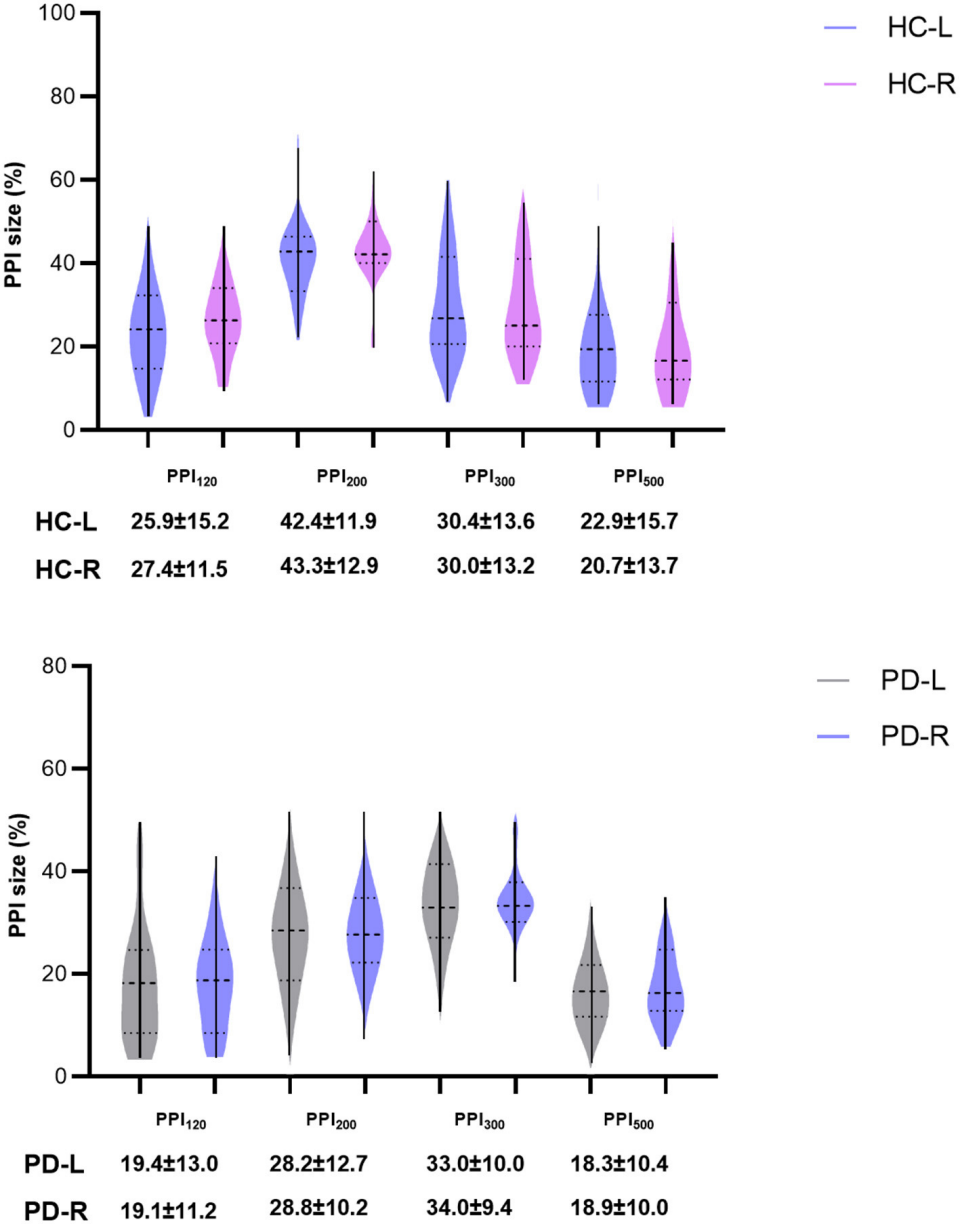
Comparisons of PPI between left and right sides of HC group and PD groups. PPI_120_, PPI_200_, PPI_300_, and PPI_500_ represent prepulse inhibition at interstimulus intervals (ISIs) of 120 ms, 200 ms, 300 ms, and 500 ms, respectively. Comparisons between two groups at each ISI were performed using the independent-sample *t*-test. All *p-*values were corrected by FDR.

While this study aimed to explore potential differences in left and right blink reflex responses to PPI across the HC and PD groups (Figure 6), due to space limitations, we focused on data derived from the left side in previous sections. Importantly, we did analyze PPI for both the left and right sides. Our findings revealed no significant statistical difference in PPI between the left and right sides within either the HC or PD groups (Figure 6). This suggests that there is no evidence of lateralization of PPI dysfunction in this study. Additionally, lacking significant difference between sides implies that the PD onset side did not influence the overall PPI results.

## 5 Discussion

This study is the first to investigate PPI dysfunction among Chinese PD patients. We found a PPI level of around 40% in the HC group, with the most significant inhibition observed at 200 ms ISI. This finding aligns with our previous research (9). It's important to consider that racial background, in addition to factors like age and gender, may contribute to result variations (18). Notably, previous international studies report the most prominent PPI in healthy populations at 120 ms (9). This difference might be due to the use of weak electrical finger stimulation in our study compared to the established auditory prepulse stimulation method employed in international research. Sensory information from the limbs travels slower to facial motor neurons and the prepulse circuit compared to auditory input (19). Additionally, the faster conduction time of auditory input to the brainstem center might lead to quicker information processing within the prepulse circuit (20).

While the auditory mode is well-established, somatosensory electrical stimulation offers a distinct advantage. Research suggests that PPI elicited by emotional prepulse stimuli (like scary images) is significantly stronger than that caused by neutral stimuli (21). Even the participants' expectations of electric shocks can enhance PPI. In this context, somatosensory prepulse stimulation provides a more objective measure of PPI dysfunction, as it minimizes the influence of emotional or expectation-based biases.

The blink reflex (BR) offers a valuable tool for assessing brainstem function. When the supraorbital nerve is stimulated, the orbicularis oculi muscle produces two key responses: the early ipsilateral component (R_1_) and the late bilateral component (R_2_). R_1_ arises from simpler medullary circuits with few synapses, while R_2_ involves more complex, multi-synaptic connections within the medulla (22). Notably, the brainstem appears to be the primary anatomical site for PPI, as this phenomenon persists even in animals lacking a brain (23). A weak prepulse stimulation applied before stimulating the supraorbital nerve can induce a blink reflex. By analyzing the R_2_ component, we can evaluate the excitability of brainstem circuits. In patients with motor disorders like Parkinson's disease (PD), heightened R_2_ component excitability represents a characteristic feature of the blink reflex (12, 24). Our findings align with this established knowledge. The observed decrease in PPI within the PD patients compared to the healthy controls (HC), alongside the increased baseline R_2_ curve area in PD patients, reinforces the connection between reduced PPI and elevated R_2_ excitability in PD.

While exact mechanisms behind the observed changes in R_2_ excitability and PPI in our PD group remain under investigation, the basal ganglia circuitry likely plays a significant role. The subthalamic nucleus (STN), a key structure within the basal ganglia, might contribute alongside other brain regions to regulating R_2_ component excitability in PD. However, it's important to note that the STN has a well-defined input-output relationship with the pontine nucleus (PPTN). The basal ganglia may exert control over the prepulse circuit, potentially regulating the excitability of brain structures involved in the startle reflex through its influence on the PPTN. Supporting this notion, lesion studies have shown that a decrease in cholinergic inhibitory neurons within the PPTN leads to an increase in startle response amplitude and a decrease in PPI (12, 25).

PPTN maintains intricate connections with several nuclei, including STN, globus pallidus interna (GPi), and substantia nigra pars reticulata (SNr). While increased excitatory input from STN to PPTN might occur, the inhibitory effect of the GPi/SNr pathway on PPTN could potentially counteract this excitation in PD patients.

However, the situation is further influenced by a reduction in the direct (D_1_) striatal projection to GPi in PD. The absence of D_1_ receptor stimulation in the GPi due to reduced striatal input may lead to a disinhibition and potential increase in GPi excitability. This heightened GPi activity could then exert a stronger inhibitory effect on PPTN compared to the excitatory influence of STN (19).

Stimulation of a single PPTN significantly affects the excitability of the brainstem neuronal circuit mediated by BR, with a significant increase in PPI (12). It can be seen that PPTN mainly affects and regulates the excitability and PPI of brainstem neuronal circuits. It's important to acknowledge the existence of other regulatory pathways for PPI, such as the cortico-striato-pallido-pontine (CSPP) circuit (26). This limbic circuit involves the prefrontal cortex, amygdala, and hippocampus influencing PPI through the nucleus accumbens. In turn, the nucleus accumbens regulates PPI by modulating the ventral pallidum and ultimately the PPTN, establishing a top-down control mechanism.

Our study found that PD patients had a longer baseline R1 latency compared to the healthy control group (HC). This suggests a potential decrease in brainstem circuit function in PD patients compared to HC. PPTN is primarily associated with functional regulation such as gait and sleep (7, 27). Interestingly, our study observed a more pronounced PPI impairment in the PD-PIGD subgroup in comparison to the PD-TD subgroup. This finding suggests that PD-PIGD patients might have poorer PPTN regulation of PPI function. However, the involvement of cortical descending pathways in this difference cannot be ruled out.

While PPTN plays a key role in regulating PPI, other factors may also influence this process in PD. For instance, PD-TD patients often require heightened attention to control limb tremors (28). Notably, attention mechanisms are emerging as important contributors to PPI function. Although PPI is considered an automated process, human experimental psychology confirms that PPI receives top-down regulation from cognitive activities such as attention and emotion (29). Studies in healthy subjects show that selective attention and cognitive processing to prepulse stimuli can significantly enhance PPI (30). Prepulse stimulation can trigger cognitive activities such as attention and emotion. These processes not only enhance the processing of prepulse information in various sensory centers but also amplify the inhibition of movement responses triggered by subsequent, interfering stimuli (like shock stimuli). This ultimately strengthens the protective effect of prepulse stimulation.

Anatomically, there are a large number of bidirectional nerve fibers in the human thalamic medullary nucleus and putamen, which are the most important connecting pathways between the thalamus and putamen and participate in attention-related information transmission. Interestingly, studies using resting-state functional magnetic resonance imaging have shown enhanced putamen-thalamic connectivity in the PD-TD subtype (28), suggesting that heightened attention is required for PD-TD patients to manage their tremor symptoms. Based on these observations, we propose that the brain's ability to filter out irrelevant information and extract target stimuli for deeper processing relies on two complementary mechanisms: the brainstem-level gating, and the forebrain-level attention.

Our study further investigated the potential link between the initial side of limb tremors (onset side) and the degree of PPI impairment (left vs. right side) in PD patients. However, similar to previous research (12, 31, 32), no statistically significant differences were found in the magnitude of left and right PPI. This finding suggests that the PPI deficits in PD are not lateralized, meaning they affect both sides of the body to a similar extent.

In addition to age, and gender, several other factors may contribute to the differentiation between PD-TD and PD-PIGD subtypes. Genetic studies have suggested that Parkinson's disease patients with LRRK2 mutations are more likely to exhibit the PIGD phenotype (33), while those who are homozygous for the H1 haplotype of the microtubule-associated protein tau (MAPT) gene tend to present with a non-tremor-dominant phenotype (34). A study (35) using quantitative susceptibility mapping (QSM) to examine iron deposition in the deep gray matter of different PD subtypes found that tremor-dominant PD patients had higher iron content in the dentate nucleus, whereas PIGD patients exhibited greater iron accumulation in both the substantia nigra pars compacta and pars reticulata. Parkinson's disease patients with different motor subtypes have differences in clinical features, genetic factors, neuroimaging and electrophysiology.

A major challenge in this study is the potential difficulty in distinguishing PD-PIGD from multiple system atrophy (MSA). Although we applied the MSA diagnostic criteria (36) to exclude MSA cases as rigorously as possible, the possibility of misclassification cannot be entirely ruled out. In future research, we plan to expand our cohort to include more patients with MSA and other movement disorders to perform PPI assessments. We will analyze whether different MSA subtypes and PD-PIGD exhibit statistically significant differences. Furthermore, we intend to explore variations in the blink reflex excitability recovery curve (19) across different movement disorders to identify potential biological markers for improved differentiation.

## 6 Conclusion

Our study found that Parkinson's disease (PD) patients, particularly those exhibiting postural instability and gait difficulty, have impaired prepulse inhibition (PPI) across various stimulation timings. This suggests abnormal brainstem circuits and potentially dysfunctional pontine nuclei (PPTN) in PD, as PPTN is known to regulate both PPI and gait. Interestingly, attention mechanisms also seem to influence PPI. In conclusion, PPI has the potential to serve as an early biomarker for gait dysfunction in Parkinson's disease, helping to predict and monitor disease progression.

## Data Availability

The original contributions presented in the study are included in the article/supplementary material, further inquiries can be directed to the corresponding authors.

## References

[1] Zhang Z Roman G Hong Z Wu C Qu Q Huang J. Parkinson's disease in China: prevalence in Beijing, Xian, and Shanghai. Lancet. (2005) 365:595–7. 10.1016/S0140-6736(05)70801-1 15708103

[2] Lees AJ Hardy J Revesz T. Parkinson's disease. Lancet. (2009) 373:2055–66. 10.1016/S0140-6736(09)60492-X 19524782

[3] Aarsland D Andersen K Larsen JP Lolk A. Prevalence and characteristics of dementia in Parkinson disease. Arch Neurol. (2003) 60:387. 10.1001/archneur.60.3.387 12633150

[4] Jankovic J Kapadia AS. Functional decline in Parkinson disease. Arch Neurol. (2001) 58:1611. 10.1001/archneur.58.10.1611 11594919

[5] Pagano G Ferrara N Brooks DJ Pavese N. Age at onset and Parkinson disease phenotype. Neurology. (2016) 86:1400–7. 10.1212/WNL.0000000000002461 26865518 PMC4831034

[6] Alves G Larsen JP Emre M Wentzel-Larsen T Aarsland D. Changes in motor subtype and risk for incident dementia in Parkinson's disease. Movement Disorders. (2006) 21:1123–30. 10.1002/mds.20897 16637023

[7] Ferraye MU Debu B Fraix V Goetz L Ardouin C Yelnik J. Effects of pedunculopontine nucleus area stimulation on gait disorders in Parkinson's disease. Brain. (2009) 133:205–14. 10.1093/brain/awp229 19773356

[8] Thevathasan W Cole MH Graepel CL Hyam JA Jenkinson N Brittain J-S. A spatiotemporal analysis of gait freezing and the impact of pedunculopontine nucleus stimulation. Brain. (2012) 135:1446–54. 10.1093/brain/aws039 22396391 PMC3338924

[9] Hao X Huang X Yin X Wang H-Y Lu R Liang Z. Elucidation of the mechanism underlying impaired sensorimotor gating in patients with primary blepharospasm using prepulse inhibition. Front Neurol. (2023) 14:1105483. 10.3389/fneur.2023.1105483 36816573 PMC9929365

[10] Geyer MA Krebs-Thomson K Braff DL Swerdlow NR. Pharmacological studies of prepulse inhibition models of sensorimotor gating deficits in schizophrenia: a decade in review. Psychopharmacology. (2001) 156:117–54. 10.1007/s002130100811 11549216

[11] Ahmari SE Risbrough VB Geyer MA Simpson HB. Impaired sensorimotor gating in unmedicated adults with obsessive–compulsive disorder. Neuropsychopharmacology. (2012) 37:1216–23. 10.1038/npp.2011.308 22218093 PMC3306882

[12] Insola A Mazzone P Della Marca G Capozzo A Vitale F Scarnati E. Pedunculopontine tegmental Nucleus-evoked prepulse inhibition of the blink reflex in Parkinson's disease. Clin Neurophysiol. (2021) 132:2729–38. 10.1016/j.clinph.2021.06.028 34417108

[13] Öztürk O Gündüz A Kiziltan ME. Deficient median nerve prepulse inhibition of the blink reflex in cervical dystonia. Clin Neurophysiol. (2016) 127:3524–8. 10.1016/j.clinph.2016.09.013 27815976

[14] Postuma RB Berg D Stern M Poewe W Olanow CW Oertel W. MDS clinical diagnostic criteria for Parkinson's disease. Movement Disor. (2015) 30:1591–601. 10.1002/mds.26424 26474316

[15] Braff DL Geyer MA Swerdlow NR. Human studies of prepulse inhibition of startle: normal subjects, patient groups, and pharmacological studies. Psychopharmacology. (2001) 156:234–58. 10.1007/s002130100810 11549226

[16] Kumari V Konstantinou J Papadopoulos A Aasen I Poon L Halari R. Evidence for a role of progesterone in menstrual cycle-related variability in prepulse inhibition in healthy young women. Neuropsychopharmacology. (2009) 35:929–37. 10.1038/npp.2009.195 19956084 PMC3055354

[17] Tomlinson CL Stowe R Patel S Rick C Gray R Clarke CE. Systematic review of levodopa dose equivalency reporting in Parkinson's disease. Movement Disor. (2010) 25:2649–53. 10.1002/mds.23429 21069833

[18] Swerdlow NR Talledo JA Braff DL. Startle modulation in Caucasian-Americans and Asian-Americans: a prelude to genetic/endophenotypic studies across the “Pacific Rim”. Psychiatr Genet. (2005) 15:61–5. 10.1097/00041444-200503000-00010 15722959

[19] Valls-Solé J Muñoz JE Valldeoriola F. Abnormalities of prepulse inhibition do not depend on blink reflex excitability: a study in Parkinson's disease and Huntington's disease. Clin Neurophysiol. (2004) 115:1527–36. 10.1016/j.clinph.2004.02.014 15203054

[20] Valls-Solé J Valldeoriola F Molinuevo JL Cossu G Nobbe F. Prepulse modulation of the startle reaction and the blink reflex in normal human subjects. Exper Brain Res. (1999) 129:49–56. 10.1007/s002210050935 10550502

[21] Bradley MM Codispoti M Lang PJ. A multi-process account of startle modulation during affective perception. Psychophysiology. (2006) 43:486–97. 10.1111/j.1469-8986.2006.00412.x 16965611

[22] Szmidt-Salkowska E Gawel M Jamrozik Z Salkowska-Wanat J Gawel D Kaminska A. Diagnostic value of blink reflex in multisystem atrophy, progressive supranuclear palsy and Parkinson disease. Neurol Neurochir Pol. (2016) 50:336–41. 10.1016/j.pjnns.2016.06.001 27591058

[23] Li L Frost BJ. Azimuthal directional sensitivity of prepulse inhibition of the pinna startle reflex in decerebrate rats. Brain Res Bull. (2000) 51:95–100. 10.1016/S0361-9230(99)00215-4 10654587

[24] Valls-Sole J. Assessment of excitability in brainstem circuits mediating the blink reflex and the startle reaction. Clin Neurophysiol. (2012) 123:13–20. 10.1016/j.clinph.2011.04.029 22030138

[25] Tapias-Espinosa C Río-Álamos C Sánchez-González A Oliveras I Sampedro-Viana D Castillo-Ruiz M. Schizophrenia-like reduced sensorimotor gating in intact inbred and outbred rats is associated with decreased medial prefrontal cortex activity and volume. Neuropsychopharmacology. (2019) 44:1975–84. 10.1038/s41386-019-0392-x 30986819 PMC6784988

[26] Fendt M Li L Yeomans JS. Brain stem circuits mediating prepulse inhibition of the startle reflex. Psychopharmacology. (2001) 156:216–24. 10.1007/s002130100794 11549224

[27] Chambers NE Lanza K Bishop C. Pedunculopontine nucleus degeneration contributes to both motor and non-motor symptoms of parkinson's disease. Front Pharmacol. (2020) 10:1494. 10.3389/fphar.2019.01494 32009944 PMC6974690

[28] Gu Q Cao H Xuan M Luo W Guan X Xu J. Increased thalamic centrality and putamen–thalamic connectivity in patients with parkinsonian resting tremor. Brain Behav. (2016) 7:e00601. 10.1002/brb3.601 28127519 PMC5256184

[29] Li L Du Y Li N Wu X Wu Y. Top–down modulation of prepulse inhibition of the startle reflex in humans and rats. Neurosci Biobehav Rev. (2009) 33:1157–67. 10.1016/j.neubiorev.2009.02.001 19747594

[30] Ashare RL Hawk LW Mazzullo RJ. Motivated attention: Incentive effects on attentional modification of prepulse inhibition. Psychophysiology. (2007) 44:839–45. 10.1111/j.1469-8986.2007.00563.x 17640265 PMC2650018

[31] Yust-Katz S Tesler D Treves TA Melamed E Djaldetti R. Handedness as a predictor of side of onset of Parkinson's disease. Parkinsonism Relat Disor. (2008) 14:633–5. 10.1016/j.parkreldis.2008.01.017 18346926

[32] Djaldetti R Ziv I Melamed E. The mystery of motor asymmetry in Parkinson's disease. The Lancet Neurology. (2006) 5:796–802. 10.1016/S1474-4422(06)70549-X 16914408

[33] Belvisi D Fabbrini A De Bartolo MI Costanzo M Manzo N Fabbrini G. The pathophysiological correlates of Parkinson's disease clinical subtypes. Movement Disorders. (2020) 36:370–9. 10.1002/mds.28321 33037859

[34] Fereshtehnejad S-M Postuma RB. Subtypes of Parkinson's disease: what do they tell us about disease progression? Curr Neurol Neurosci Rep. (2017) 17:34. 10.1007/s11910-017-0738-x 28324303

[35] Armstrong MJ Okun MS. Diagnosis and treatment of Parkinson disease. JAMA. (2020) 323:548. 10.1001/jama.2019.22360 32044947

[36] Wenning GK Stankovic I Vignatelli L Fanciulli A Calandra-Buonaura G Seppi K. The movement disorder society criteria for the diagnosis of multiple system atrophy. Movement Disorders. (2022) 37:1131–48. 10.1002/mds.29005 35445419 PMC9321158

